# Copigmentation effect of three phenolic acids on color and thermal stability of Chinese bayberry anthocyanins

**DOI:** 10.1002/fsn3.1583

**Published:** 2020-05-20

**Authors:** Yanyun Zhu, Hongji Chen, Leyan Lou, Yixin Chen, Xingqian Ye, Jianchu Chen

**Affiliations:** ^1^ College of Biosystems Engineering and Food Science National‐Local Joint Engineering Laboratory of Intelligent Food Technology and Equipment Zhejiang Key Laboratory for Agro‐Food Processing Zhejiang Engineering Laboratory of Food Technology and Equipment Zhejiang University Hangzhou China; ^2^ Ningbo Research Institute Zhejiang University Ningbo China

**Keywords:** Bayberry anthocyanins, copigmentation, phenolic acids, thermodynamic parameters

## Abstract

The copigmentation effects of three phenolic acids (ferulic acid, sinapic acid, and syringic acid) on the color and thermal stability of Chinese bayberry anthocyanins were investigated. The three copigments of ferulic acid, sinapic acid, and syringic acid were found to have significant effects on the color enhancement of bayberry anthocyanins (*p* < .05). The maximum absorption wavelength of the anthocyanin aqueous solution exhibited a bathochromic shift, L* decreased, and a* increased with the increase in the molar ratio of copigments. The thermal stability of bayberry anthocyanins was significantly improved after copigmentation. No new anthocyanin derivatives appeared after copigmentation. The thermodynamic parameters (Δ*G*, Δ*H*, Δ*S*) of the three processes were all negative, indicating the three copigmentations were generally spontaneous and exothermic. The equilibrium constant (*K*) of the sinapic acid group was the greatest among the three phenolic acids, indicating that sinapic acid was more effective in stabilizing the anthocyanins.

## Graphical abstract

The objective of this study was to investigate the detailed copigmentation abilities of three common phenolic acids (ferulic acid [FeA], sinapic acid [SiA] and syringic acid [SyA]) on colour and thermal stability of Chinese bayberry anthocyanins.

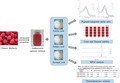

## 1 INTRODUCTION

Chinese bayberry (*Myrica rubra* Sieb. et Zucc.) is a subtropical plant native to China, with high economic and health‐promoting value (Yu, Zhang, Zhao, & Tian, 2018). Bayberries are a popular fruit among consumers due to the delicious taste and attractive color, but the fruit is usually processed, for example, into juice, wine, and jam, due to its intense postharvest respiration and poor preservation technology (Fang, Zhang, Tao, Sun, & Sun, 2006).

Anthocyanins are crucial pigments responsible for the attractive colors of red bayberry juice and wine. As a natural source of pigments, anthocyanins have attracted great interest in recent years because of their related health‐promoting benefits, including antioxidant, anti‐inflammatory, and chemoprotective properties (He & Giusti, 2010; Howard, Castrodale, Brownmiller, & Mauromoustakos, 2010; Özgen, Saraçoğlu, & Geçer, 2012; Qin et al., 2009; Sancho & Pastore, 2012). However, anthocyanins are highly unstable and readily susceptible to degradation by factors, such as temperature, light, pH, solvents, and oxygen exposure (Howard et al., 2010; Jimenez et al., 2010; Malaj, Simone, Quartarolo, & Russo, 2013). Accordingly, it is essential but challenging to protect anthocyanins from degradation.

Previous studies found that red berry‐processed products still display an intense red color in these unstable circumstances, and copigmentation was identified as one of the factors responsible for the color retention (Boulton, 2001; Lorenzo, Pardo, Zalacain, Alonso, & Salinas, 2005). Four types of copigmentation mechanisms have been identified, including intramolecular copigmentations, intermolecular copigmentations, self‐association, and metal complexation. Among them, intramolecular and intermolecular copigmentations are the two most important types existing in nature, considered as the potential factors that might affect the stability of the anthocyanin (Boulton, 2001; Czibulya, Horváth, Kollár, & Kunsági‐Máté, 2012; Sharara, 2017). The copigmentation effect is manifested by an enhancement in the color intensity of the solution and a change in color tonality when an aqueous solution of pigment (anthocyanin), in slightly acidic media, is added to a colorless copigment. The effect causes an increase of absorbance in the visible range (hyperchromic shift) and a shift of the wavelength of the maximum absorbance toward higher values (bathochromic shift). Both can be detected spectrophotometrically (Dangles & Brouillard, 1992; Lambert, Asenstorfer, Williamson, Iland, & Jones, 2011; Markovic, Petranović, & Baranac, 2005). In recent years, the quantum mechanics calculation has been employed by many researchers to study the copigmentation (Di Meo, Sancho Garcia, Dangles, & Trouillas, 2012), which disclosed the copigmentation mechanism from the microperspective, including the electron and charge transfer, thermodynamic parameters (Teixeira et al., 2013), intermolecular bonding type, and others (Kalisz, Oszmiański, Hładyszowski, & Mitek, 2013; Zhang, Liu, He, Zhou, & Duan, 2015).

Phenolic acids play an important role in enhancing and stabilizing the color of various fruits and vegetables, indicating that they are capable of acting as good copigments with anthocyanins (Malaj et al., 2013; Sharara, 2017; Xu, Liu, Yan, Yuan, & Gao, 2015). However, the copigmentation between phenolic acids and bayberry anthocyanins has not yet been reported. Hence, it is meaningful to assess the color changes and color‐developing features of the copigmentation between phenolic acids and bayberry anthocyanin. In this study, we aim to investigate the detailed copigmentation abilities of three common phenolic acids (ferulic acid [FeA], sinapic acid [SiA], and syringic acid [SyA]) in bayberry anthocyanin solutions. Thermodynamic data and high‐performance liquid chromatography (HPLC) analysis were used to improve the understanding of the mechanism of the intermolecular copigmentation between the phenolic acids and anthocyanins.

## 2 MATERIALS AND METHODS

### 2.1 Materials

Chinese bayberry (M. rubra) was grown in the Cixi area of Zhejiang Province, China. The phenolic acids FeA (99%), SiA (98%), and SyA (98%) were purchased from Aladdin All other chemicals used were of analytical grade. The solutions were prepared using distilled–deionized water.

### 2.2 Extraction of anthocyanins

The Chinese bayberry anthocyanins were extracted using the modified method described by Liu, Gao, Jiang, Yang, and Sun (2014). Fifty grams of freeze‐dried bayberry was added to 500 ml of 60% ethanol solution containing 0.1% HCl, sonicated for 3 hr, filtered, and the filter residue then ultrasonically combined with 500 ml of 65% ethanol solution containing 0.1% HCl for 3 hr, filtered and combined with the resulting filtrates. The clarified solution of anthocyanins was then concentrated by rotary evaporation (<40°C) to about 50 ml, and the ethanol was removed. The concentrated solution of anthocyanins was kept away from light at −4℃, for use.

### 2.3 Heating processing experiments

The sample solution of anthocyanins was diluted 200‐fold with a 0.1 M pH 3.4 sodium dihydrogen phosphate buffer solution. The following two operations were then carried out:

#### 2.3.1 Procedure 1

To examine the effect of the pigment/copigment molar ratio on copigmentation, solutions were prepared by mixing an equal volume of the anthocyanin sample solution with the three phenolic acids (copigments), respectively, to obtain the ratios of 1:0, 1:1, 1:5, 1:10, 1:20, and 1:30. The tubes were then placed in a water bath at 80°C for 2 hr.

#### 2.3.2 Procedure 2

To investigate the effect of copigmentation on the thermal stability, an equal volume of anthocyanin sample solution was mixed with the three phenolic acids (copigments), respectively, to give the required 1:20 pigment/copigment molar ratio in the tubes. Anthocyanins are extremely unstable at high temperatures. Anthocyanins do not fade very quickly at temperatures of 70 80 and 90℃, and can be used to evaluate the thermal stability of anthocyanins. And so the tubes were then placed in a water bath at 70, 80, and 90°C for 5 hr. Samples were collected at 1‐hr intervals and cooled down in an ice–water bath.

### 2.4 Copigmentation and colorimetric measurement

The whole visible spectrum (400–700 nm) was recorded at the constant intervals of Δλ = 1 nm, using a UV‐2550 UV–Visible (UV–Vis) spectrophotometer (Shimadzu Co.), 10‐mm path‐length glass cells and distilled water as a reference. The magnitudes of the hyperchromic effect and bathochromic shift in the maximum absorbance were reported as (*A* − *A*_0_)/*A*_0_ and ∆λ_max_ = λ_max_ − λ_512_, respectively (*A*_0_ was the maximum visible absorbance at λ = 512 nm for the anthocyanin sample solution in the absence of the copigments, *A* was the absorbance at λ_512_ of the anthocyanin sample solution in the presence of the copigments, and λ_max_ was the wavelength of maximum absorbance) (Zhang et al., 2015).

The CIELab colorimetric space was used to characterize the change in color (Xu et al., 2015) using a HunterLab ColorFlex EZ automatic chromatic meter (HunterLab Co.). The color parameters L* (lightness), a* (green/red component), and b* (blue/yellow component) were recorded directly. In order to visualize the color changes, color swatches were created from the L*, a*, and b* values of each sample using Adobe Photoshop CC software (Adobe Systems, Inc.) (Sui, Bary, & Zhou, 2016).

### 2.5 Thermodynamic data determinations

The energy information can be provided by the thermodynamic data, which can help to understand the mechanism of the copigmentation process. The equilibrium constant (*K*) of the copigmentation reaction quantified the strength of the association between the copigment and the pigment (Brouillard et al., 1988). Thermodynamic data of pigment/copigment systems were calculated by the modified method of Malaj et al. (2013). The equilibrium constant (*K*), Gibbs free energy (Δ*G*), enthalpy change (Δ*H*), and entropy change (Δ*S*) of the copigmentation reaction were determined by the following equations ((1), (2), (3), (4)):

(1) $$\ln\left(\frac{A-A_{0}}{A_{0}}\right)=\ln\left(K\right)+n\ln{\left[\text{CP}\right]}_{0}$$

(2) ΔG=-RTlnK

(3) $$\ln\left(\frac{A-A_{0}}{A_{0}}\right)=-\frac{\Delta H}{\text{RT}}$$

(4) ΔG=ΔH-TΔS

where *A* and *A*_0_ were the absorbance at λ_512_ of the anthocyanin solution after and before mixed copigment, respectively; *n* was the stoichiometric ratio between the anthocyanin and the copigment; [*CP*]_0_ represented the concentration of copigment added to the anthocyanin solution; *R* was the gas constant (8.314 J mol K^−1^); and *T* was the temperature in Kelvin (10^–3^ K^‐1^).

### 2.6 HPLC analysis of anthocyanins

The anthocyanins were analyzed, according to Tiwari, Patras, Brunton, Cullen, and O'Donnell (2010). A reversed‐phase column (Agilent Zorbax SB C18, 250 × 4.6 mm, 5 μm; Agilent Technologies) was used. The eluent solvents were 5% (v/v) formic acid (solvent A) and 100% (v/v) methyl alcohol (solvent B). A gradient elution program was applied as follows: (a) 5% B in 5 min, (b) from 5% to 25% B in 5–8 min, (c) from 25% to 30% B in 8–15 min, and (d) from 30% to 25% B in 15–20 min. The detection wavelength was 520 nm; the flow rate was 1 ml/min; the column temperature was maintained at 30°C; and the injection volume was 10 μl.

## 3 RESULTS AND DISCUSSION

### 3.1 Effect of pigment/copigment molar ratio on the copigmentation

**Figure 1:**
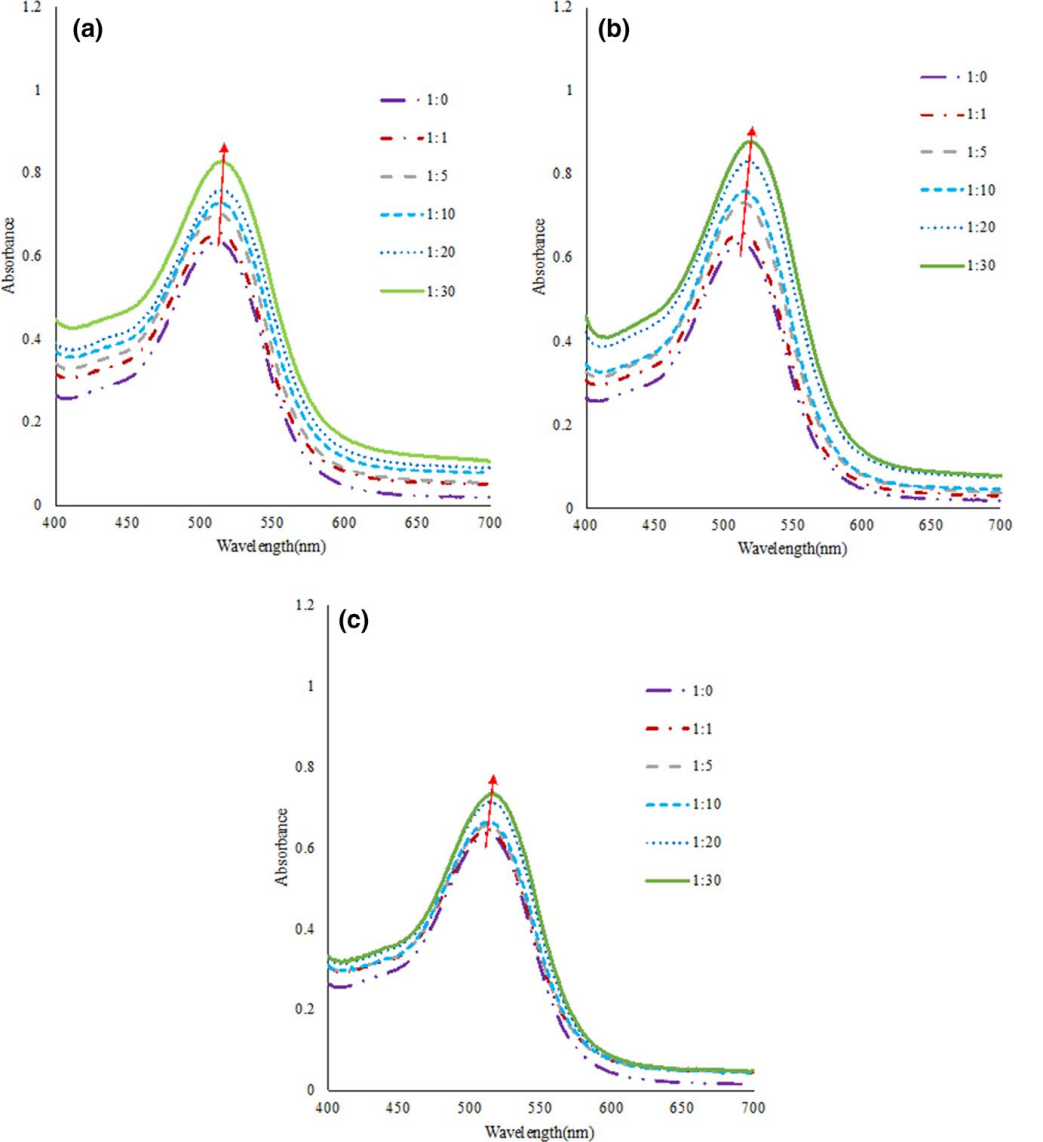
The UV–Vis of bayberry anthocyanin solution when added with different molar of copigments before heat treatment （a. ferulic acid （FeA), b. sinapic acid （SiA), and c. syringic acid [SyA])

The concentration of anthocyanin extract was kept constant, and its copigmentation with increasing amounts of copigments involved (anthocyanin extract/copigments molar ratios of 1:1, 1:5, 1:10, 1:20, and 1:30) was examined in a model solution (pH 3.4). As shown in Figure 1 and Table 1, the addition of increasing levels of the copigments to a constant anthocyanin concentration produced a bathochromic shift and an increasing absorbance of solutions over all the visible range, indicating the existence of a copigmentation process. In addition, as the concentration of the copigments increased, the copigment effect became more obvious. The strongest immediate copigmentation effect at the 1:30 molar ratio occurred between the anthocyanins and each of the three phenolic acids, showing the most dramatic hyperchromic effect and the largest bathochromic shift, which was also supported by Brouillard, Mazza, Saad, Albrecht‐Gary, and Cheminat (1988), who stated that intermolecular copigmentations are often formed at high mole ratios.

**Table 1:** L*, a*, and b* values of bayberry anthocyanin solution when added with different molar of copigments before heat treatment

Color parameter	Molar ratio	FeA/anthocyanins		SiA/anthocyanins		SyA/anthocyanins	
		Values	Delta *E*	Values	Delta *E*	Values	Delta *E*
L*	1:0	43.56 ± 0.40^a^	–	43.56 ± 0.40^a^	–	43.56 ± 0.40^a^	–
	1:1	43.76 ± 0.12^a^	0.20	42.75 ± 0.12^b^	−0.82	43.28 ± 0.06^a^	−0.28
	1:5	42.67 ± 0.16^b^	−0.89	41.31 ± 0.15^c^	−2.26	42.22 ± 0.07^b^	−1.34
	1:10	42.45 ± 0.48^b^	−1.11	39.33 ± 0.04^d^	−4.24	41.47 ± 0.03^c^	−2.09
	1:20	40.98 ± 0.02^c^	−2.59	37.79 ± 0.45^e^	−5.78	40.52 ± 0.02^d^	−3.05
	1:30	39.95 ± 0.13^d^	−3.61	37.66 ± 0.45^e^	−5.91	39.16 ± 0.15^e^	−4.41
a*	1:0	41.78 ± 0.56^d^	–	41.78 ± 0.56^d^	–	41.78 ± 0.56^d^	–
	1:1	41.58 ± 0.11^d^	−0.20	43.02 ± 0.30^d^	1.24	43.34 ± 0.03^c^	1.56
	1:5	43.26 ± 0.34^c^	1.48	44.30 ± 0.69^c^	2.52	43.60 ± 0.30^c^	1.82
	1:10	43.31 ± 0.56^c^	1.53	47.01 ± 0.35^b^	5.23	44.39 ± 0.13^b^	2.61
	1:20	44.72 ± 0.13^b^	2.94	48.79 ± 0.26^a^	7.01	44.91 ± 0.11^ab^	3.13
	1:30	45.92 ± 0.14^a^	4.14	49.36 ± 0.71^a^	7.58	45.44 ± 0.08^a^	3.66
b*	1:0	26.43 ± 0.88^ab^	–	26.43 ± 0.88^ab^	–	26.43 ± 0.88^a^	–
	1:1	25.86 ± 0.09^b^c	−0.57	27.83 ± 0.21^a^	1.40	25.76 ± 0.86^ab^	−0.68
	1:5	27.29 ± 0.74^a^	0.86	25.60 ± 0.42^b^	−0.84	26.31 ± 0.01^a^	−0.12
	1:10	25.24 ± 0.68^bc^	−1.19	26.41 ± 0.61^ab^	−0.02	26.55 ± 0.14^a^	0.12
	1:20	24.90 ± 0.19^c^	−1.54	24.98 ± 1.35^b^	−1.46	25.38 ± 0.01^ab^	−1.05
	1:30	24.61 ± 0.18^c^	−1.82	22.85 ± 0.45^c^	−3.58	24.70 ± 0.15^b^	−1.74

Note

Different letters denote a significant difference at 95% confidence level by the LSD test.

Among these three copigments, SiA showed the most dramatic copigmentation effect, exhibiting the greater hyperchromic effects in comparison with the other two copigments, and bathochromic shifts of 7 nm at the molar ratio of 1:30. The *A*_λmax_ of the anthocyanins added with SiA increased significantly at the molar ratio of 1:1 relative to the control (*p* < .05), which showed that SiA could produce a significant copigmentation effect at lower concentrations than FeA and SyA. At the molar ratio of 1:5, the *A*_λmax_ of the FeA/anthocyanin complex showed a significant difference compared with the control as well (*p* < .05). However, the copigmentation effect of SyA was the lowest, and it did not show a significant difference in comparison with the control until the molar ratio reached 1:20 (*p* < .05). The anthocyanin solutions which after three phenolic acids’ copigmentation all showed red shift phenomenon, indicating that all three phenolic acids could enhance the coloration of anthocyanins, and the greater the concentration, the more obvious the color enhancement effect. May be due to the copigmentation of phenolic acids in intramolecular and intermolecular.

As can be seen in Figure 1, the copigmentation phenomena induced by the three phenolic acids did not only increase *A*_512_ but also altered the *λ*_max_ in the visible absorption spectrum. Therefore, to evaluate the effect of the copigmentation processes on the chromatic characteristic, the colorimetric analyses in the CIELAB space were determined (Sui et al., 2016). According to the data in Table 1, the lightness (L*) of the anthocyanin extract solution moved toward lower values, whereas coordinate a* diminished with the increase in the molar ratio, indicating that the solutions displayed a darker and more vivid red–orange. Furthermore, although the changes in the color parameters showed similar trends among the copigments, the ability of SiA to combine with bayberry anthocyanins was stronger than that of FeA and SyA under the assay conditions. For instance, from 1:1 to 1:30 molar ratio, the SiA/anthocyanin complex decreased the L* from 42.75 to 37.66 (−0.12 to −0.45 a.u.), whereas a* increased from 43.02 to 49.36 (−0.30 to −0.71 a.u.). However, these changes were only 43.76 to 39.95 (−0.12 to −0.13 a.u.) and 41.58 to 45.92 (−0.11 to −0.14 a.u.) for FeA, and only 43.28 to 39.16 (−0.06 to −0.15 a.u.) and 43.34 to 45.44 (−0.03 to −0.08 a.u.) for SyA, respectively.

### 3.2 Effect of copigmentation on thermal stability of bayberry anthocyanins

The light stability, especially UV stability, of anthocyanins, is another important characteristic for their application in food products (Bąkowska et al., 2003). To evaluate the effect of copigmentation on the thermal stability of the anthocyanins, the anthocyanin extract solutions added with the three phenolic acids, respectively, were heated at 70, 80, and 90°C for 5 hr, and *A*_512_, L*, a*, and b* were measured every hour.

**Figure 2:**
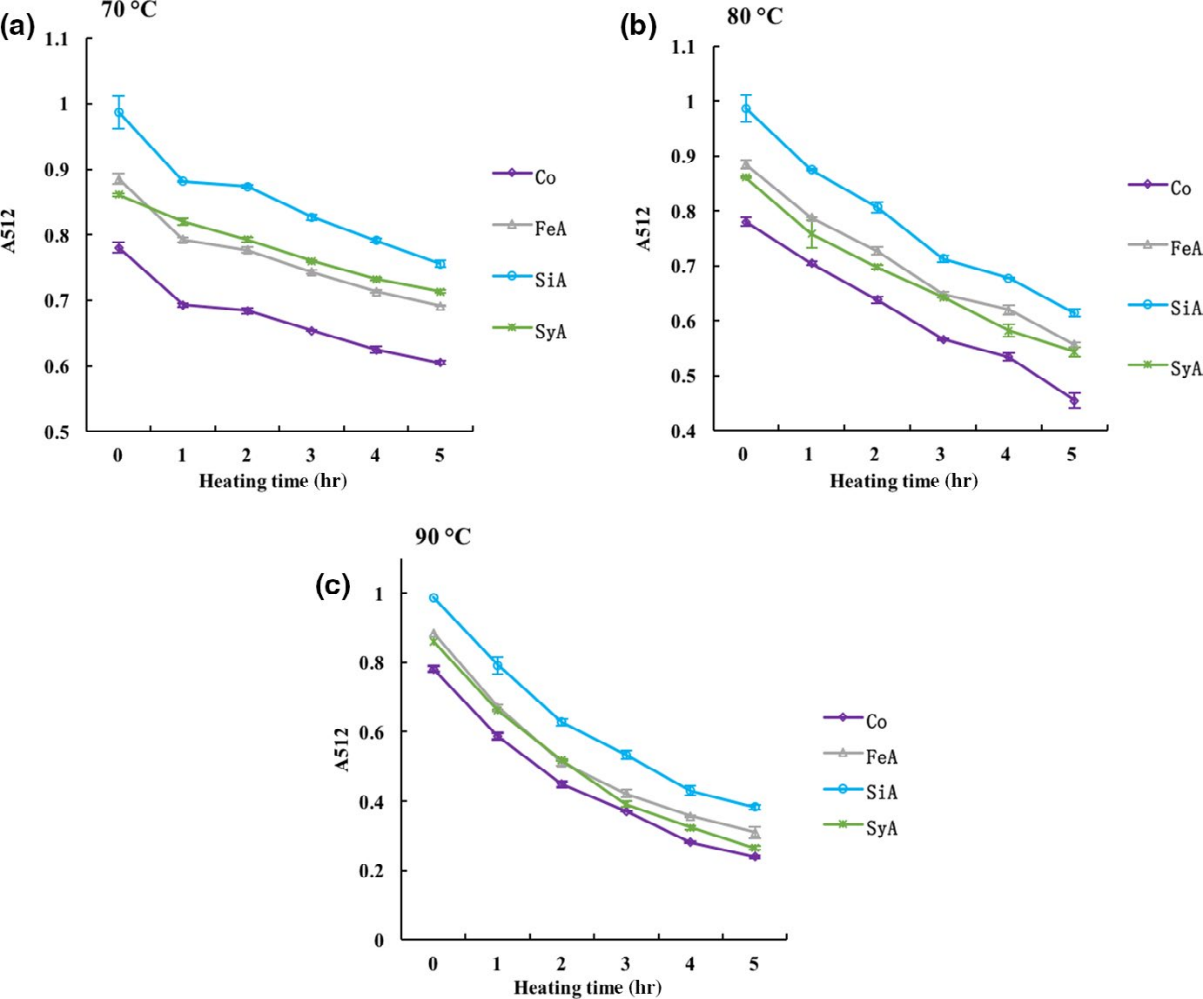
The *A*_512_ values of bayberry anthocyanin solution after copigmentation changes with the heat treatment time

As shown in Figure 2, all the pigment/copigment complexes displayed a downward trend. The higher the temperature, the faster the rate of decline, which was similar to the much lower degradation rates at lower temperatures (Sui, Dong, & Zhou, 2014). The absorbance of the control samples decreased to 0.605, 0.455, and 0.240 after being heated at 70, 80, and 90°C for 5 hr, and compared with the sample before heating, the values declined by 22.5%, 41.7%, and 69.3%, respectively. With regard to the changes in the contribution of the copigmentation to the thermal stability, the different copigments added caused different degrees of improvement in the magnitude of the effect. For example, the absorbance of FeA, SiA, and SyA decreased by 65.0%, 61.2%, and 69.2%, respectively. Among them, the absorbance of FeA and SiA decreased significantly less than the control group (*p* < .05). Furthermore, it was possible to observe that SiA had the best effect on the thermal stability of the anthocyanins. Perhaps it is because SiA has both methyl group and acrylic structure.

**Figure 3:**
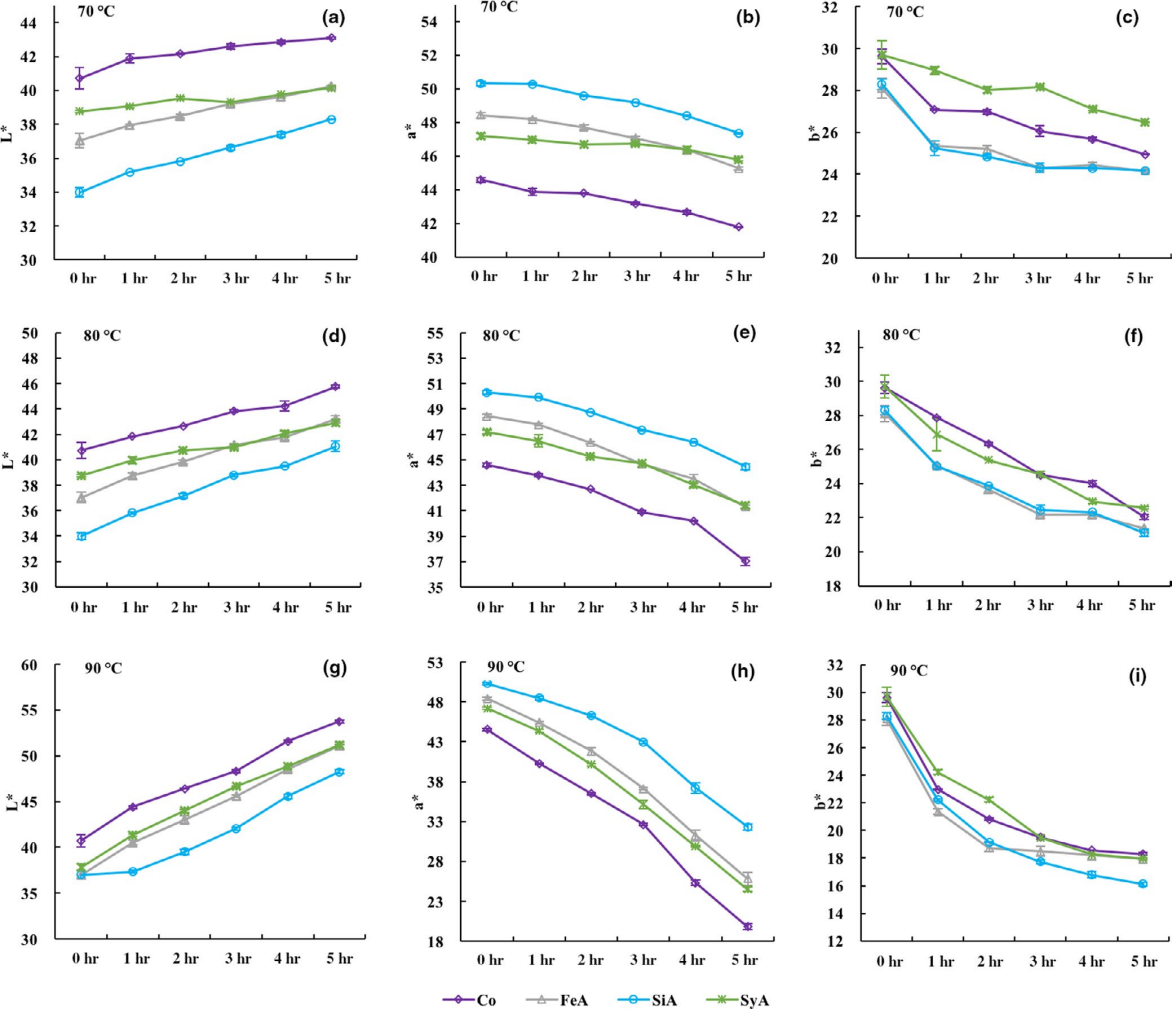
The L*, a*, and b*values of bayberry anthocyanin solution after copigmentation changes with the heat treatment time

As presented in Figure 3, during the heat treatment, the anthocyanin solution showed an upward trend in lightness (*L) with the prolongation of heating time, and a* and b* showed a downward trend, indicating that the solution became lighter in color and the redness decreased under the heating conditions. After being heated at 70, 80, and 90°C, respectively, for 5 hr, a* declined at a faster rate in the control sample, followed by the FeA and SyA samples, yet the SiA sample had the slowest rate of decline in this parameter. In addition, the control, FeA, SiA, and SyA showed a decrease in a* from 44.58, 48.43, 50.31, and 47.19 to 19.84, 25.84, 32.32, and 24.51 after heating at 90°C for 5 hr, respectively. These results were consistent with the stability enhancement of *A*_512_ with copigmentation during the thermal tests. It was in accordance with the results reported by Xu et al. (2015) that the anthocyanin stability was increased due to the copigmentation of phenolic acids.

**Figure 4:**
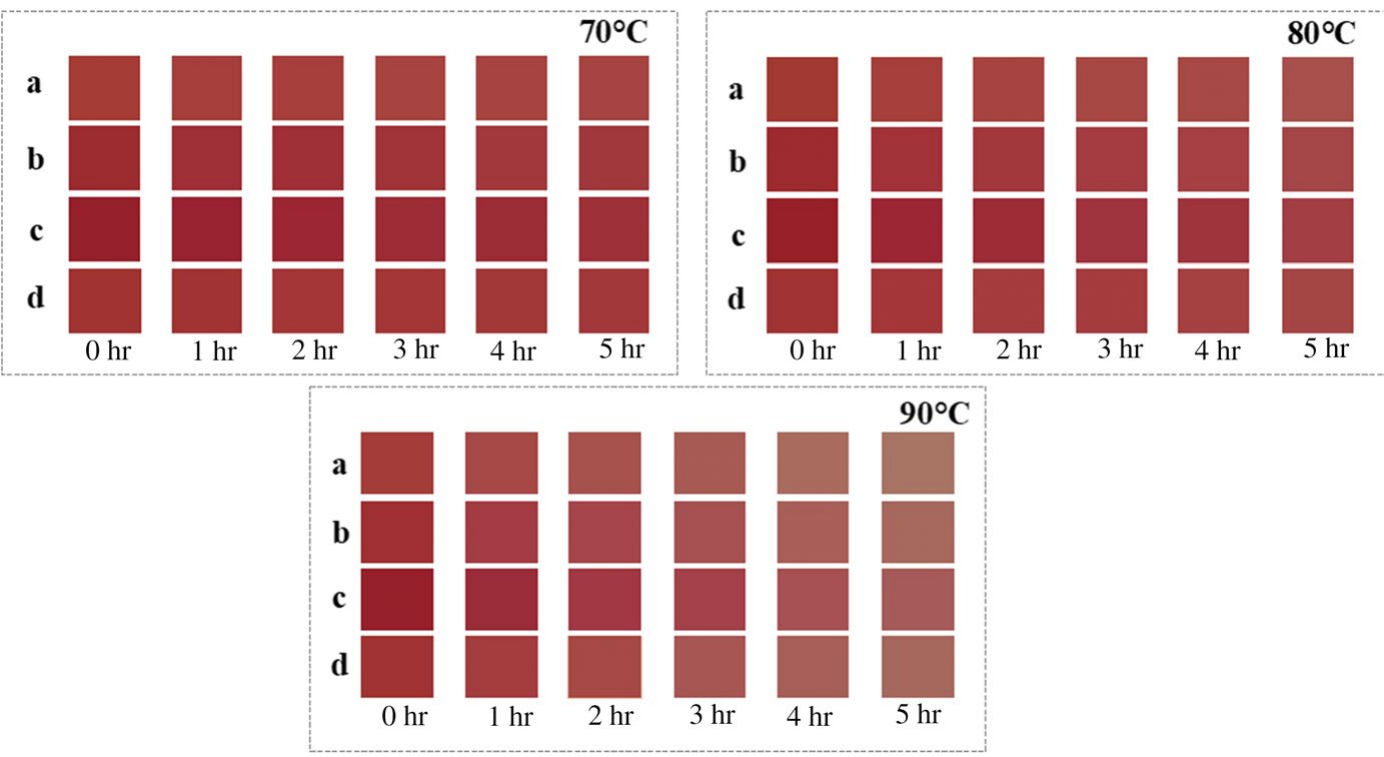
Color swatches of bayberry anthocyanin solution after copigmentation changes with the heat treatment time (**a–d** correspond to Co, FeA, SiA, and SyA, respectively)

In order to express the difference in the thermal stability between the anthocyanins with and without copigmentation, the color swatches (Figure 4) were created from L*, a*, and b* values of each sample using Adobe Photoshop CC software (Sui et al., 2016). Color differences between anthocyanin solutions copigmented with and without phenolic acids could be easily detectable by the naked eye since the color of the bayberry anthocyanin solution became relatively lighter, the redness decreased, the brownness increased with heating, and the color changed more dramatically at 90°C compared with 70 and 80°C. It was also observed that the anthocyanins added with copigments showed more orange or red–blue tonalities than the reference solution after heating, especially SiA.

### 3.3 Analysis of components before and after copigmentation of anthocyanins

**Figure 5:**
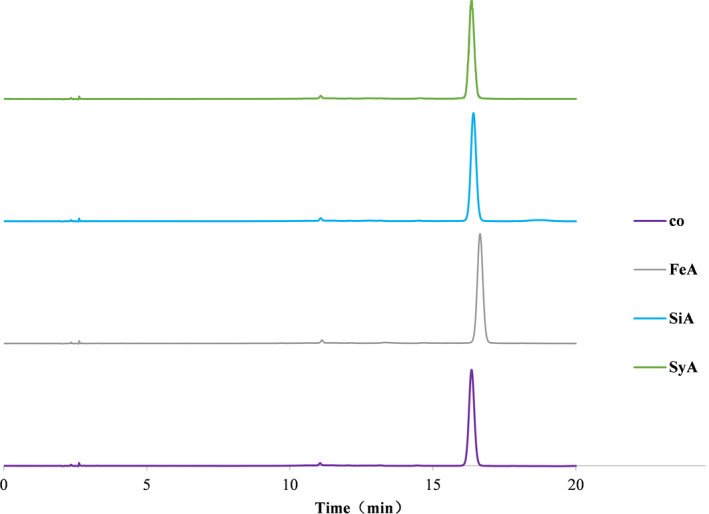
HPLC profiles of bayberry anthocyanins before and after copigmentation before heat treatment. λ = 520 nm

The liquid chromatograms of the bayberry anthocyanins before and after copigmentation are shown in Figure 5. There were no derivatives in the control sample and the three copigmentation samples, which was consistent with the anthocyanin components of the control group. According to previous reports (Bakowska‐Barczak, 2005; Tian, Deng, Wei, & Liu, 2012), the organic acid can be acylated with the sugar chain on the anthocyanin to form an acylated anthocyanin, thereby effectively protecting the cation of the anthocyanin nucleus from the nucleophilic attack of the water molecule, and improving the stability of the anthocyanin. Zhang et al. (2015) studied the copigmentation of polyphenols on mallow pigment‐3‐glucoside in red wine simulated solution, and it was assumed by quantum mechanical calculation that the secondary color interaction between the two is the result of noncovalent binding, because the π–π stack forms a more stable complex to improve the stability of anthocyanins. In our study, the anthocyanin composition did not change before and after the copigmentation, indicating that the anthocyanins did not degrade and produce new compositions. Accordingly, it was speculated that the copigmentation of sinapic acid, ferulic acid, and syringic acid and anthocyanin may also be noncovalent copigmentation.

### 3.4 Thermodynamic data for the copigmentation

As shown in Table 2, *K* varied with the copigment/pigment systems and indicated that SiA was preferred in combination with anthocyanins, followed by FeA and SyA. The stoichiometric constants (*n*) were determined from the slope of the plot ln[(*A* − *A*_0_)/*A*_0_] versus ln[*CP*]_0_, which describes the association between the copigment and the pigment. The values of *n* were approximately 0.7 or 0.8 (Table 3), suggesting that these intermolecular copigmentation reactions were relevant at a 1:1.3 ratio, which was different to the copigmentation reactions at the 1:1 ratio in the research by Lambert et al. (2011), possibly because of the difference in the anthocyanin species, polyphenols component, reaction system, etc.

**Table 2:** The equilibrium constant and thermodynamic parameters related to the process of copigmentation between copigments and bayberry anthocyanin after heat treatment

Copigment complex	*n*	*K*	Δ*G* (kJ/mol)	Δ*H *(kJ/mol)	Δ*S* (J·K^−1^·mol^−1^)
FeA/anthocyanins	0.67	17.18	−7.04	−17.90	−30.74
SiA/anthocyanins	0.68	24.80	−7.96	−15.90	−22.51
SyA/anthocyanins	0.79	16.39	−6.93	−23.38	−46.61

**Table 3:** λ_max_ and A_λmax_ of bayberry anthocyanin solutions when added with different molar of copigments before heat treatment

	Molar ratio (pigment/copigments)	FeA/anthocyanins	SiA/anthocyanins	SyA/anthocyanins
λ_max_(nm)	1:0	512	512	512
	1:1	512	513	512
	1:5	513	515	513
	1:10	514	515	513
	1:20	517	518	515
	1:30	517	519	516
A_λmax_	1:0	0.639 ± 0.011^d^	0.639 ± 0.011^d^	0.639 ± 0.011^b^
	1:1	0.659 ± 0.014^d^	0.733 ± 0.016^c^	0.639 ± 0.001^b^
	1:5	0.717 ± 0.011^c^	0.753 ± 0.018^c^	0.641 ± 0.013^b^
	1:10	0.729 ± 0.001^c^	0.781 ± 0.008^bc^	0.653 ± 0.023^b^
	1:20	0.761 ± 0.001^b^	0.832 ± 0.040^ab^	0.721 ± 0.016^a^
	1:30	0.830 ± 0.001^a^	0.878 ± 0.017^a^	0.747 ± 0.025^a^

Note

Different letters denote a significant difference at 95% confidence level by the LSD test.

The negative values of ∆*G* indicated that the three copigmentations were all spontaneous reactions. Compared with SyA (*∆G* = −6.93 kJ/mol), the highest negative value of ∆*G* obtained was for SiA (−7.96 kJ/mol), inferring that it had the best ability to combine with bayberry anthocyanins. SiA has one more methoxy group than FeA, and more acrylic structure than SyA, and the specific structure and electronic property would be the reason why the SiA had the best copigmentation. Additionally, the temperature had a key influence on the stability of the copigment/pigment complex, leading to a progressive decrease in the absorbance when the temperature was increased (Baranac, Petranović, & Dimitrić‐Marković, 1996; Malaj et al., 2013). The increased temperature tended to dissociate the copigmentation complex, probably by causing the conversion of more free colored forms, like flavylium cations, into colorless species, and thereby a loss of color. As shown in Table 2, ∆*H* was negative for all of these copigment/pigment complexes, which indicated that the process of the interaction was exothermic.

## 4 CONCLUSIONS

Three phenolic acids (FeA, SiA, and SyA) gave rise to the copigmentation interactions with the bayberry anthocyanins in the model solutions. The color parameters showed that the increasing concentration of these copigments led to increases in the solutions' absorbances (hyperchromic effect and bathochromic shift). In comparison with FeA and SyA, SiA interacted with bayberry anthocyanins to form more stable complexes, leading to the greatest copigmentation effect on the red bayberry extract model solutions. Furthermore, the equilibrium constant (*K*) and thermodynamic parameters (*∆G*, *∆H,* and *∆S*) confirmed that the SiA/anthocyanin complex was the most stable one among the phenolic acids tested, which could account for the chromatic differentiation caused by the different copigments in the visible range. Overall, the present findings are highly valuable for promoting the stability of bayberry anthocyanins in an aqueous solution system. However, the formation mechanism of phenolic acid/anthocyanin complexes still needs further investigation.

## CONFLICT OF INTEREST

The authors declare that they have no conflict of interest.
